# Assessment of histotripsy as a bone-sparing tumor ablation technique in *ex vivo* osteosarcoma tumor-affected limbs

**DOI:** 10.3389/fvets.2025.1652469

**Published:** 2026-01-09

**Authors:** Preeya F. Achari, Elliana Vickers, Lauren Ruger, Eli Vlaisavljevich, Joanne Tuohy, Caitlyn J. Collins

**Affiliations:** 1 Department of Biomedical Engineering and Mechanics, Virginia Tech, Blacksburg, VA, United States; 2 Translational Biology, Medicine, and Health, Virginia Tech, Roanoke, VA, United States; 3 Virginia-Maryland College of Veterinary Medicine, Blacksburg, VA, United States; 4 Virginia Tech Animal Cancer Care and Research Center, Roanoke, VA, United States

**Keywords:** histotripsy, osteosarcoma, bone, tumor, ablation, biomechanics, μCT

## Abstract

**Background:**

Osteosarcoma (OS) is an aggressive primary bone cancer that is highly resistant to conventional therapies. Histotripsy, a non-invasive and non-thermal focused ultrasound ablation technique, has recently been explored as a treatment for OS in a canine comparative anatomy model exhibiting heterogeneous tumor phenotypes. However, the biomechanical effects of histotripsy on OS tumor-bearing bone and adjacent grossly normal bone remain unknown. This study aimed to evaluate the impact of histotripsy on the mechanical properties of bone and to characterize tumor heterogeneity.

**Methods:**

*Ex vivo* limbs from canine OS patients (*n* = 10) were used to collect tumor-affected and normal bone specimens. Histotripsy ablation was performed on both tissue types, with corresponding unablated tumor and normal controls. Mechanical testing, including uniaxial compression and three-point bending, was conducted to determine elastic and post-yield properties. Micro-computed tomography (μCT) was used to quantify structural parameters and assess tumor heterogeneity in unablated tumor specimens. Mechanical testing revealed significantly higher elastic and post-yield properties in normal bone compared with tumor-affected bone.

**Results:**

No significant differences were detected between ablated and unablated conditions within normal bone or within tumor-affected bone, indicating that histotripsy did not compromise the structural integrity of normal bone nor exacerbate damage in tumor-affected bone. Unablated trabecular tumor specimens exhibited higher and more variable elastic modulus and ultimate strength values than other groups, highlighting tumor heterogeneity. μCT analysis confirmed substantial structural heterogeneity among unablated tumor specimens, particularly in bone volume fraction.

**Conclusion:**

Histotripsy preserves the mechanical integrity of both tumor-affected and normal bone. These results support histotripsy’s potential as a non-invasive, bone-sparing, patient-specific therapeutic approach for clinical osteosarcoma.

## Introduction

Osteosarcoma (OS) is an aggressive bone cancer that represents the most common bone malignancy in children and adolescents (1, 2). It accounts for <1% of all cancers in humans and has an incidence rate of 1.2/100,000 cases/year (2–4). The 5-year survival rate for patients with primary OS is approximately 75%; however, this rate drops dramatically to 30–35% for those who present with pulmonary metastases. This significant reduction highlights the challenge posed by metastatic disease, which has remained largely resistant to therapy and has contributed to the stagnation in survival rates over the past decades (1, 3, 5). The current standard of treatment involves a combination of chemotherapy and surgical resection; however, some patients may not qualify for surgical resection due to tumor location, insufficient treatment margins, involvement with critical structures, or presence of metastatic disease (5, 6). Patients who receive standard treatment may still develop metastasis. Surgical treatment to resect the primary tumor is associated with potential complications and co-morbidities including infection and reduced limb mobility and function (5, 7, 8). Non-surgical limb salvage options for treating the primary tumor remain extremely limited.

Canines have previously been used in osteological studies as comparative animal models. Studies have shown that adult dogs exhibit bone remodeling and age-related changes that are analogous to those present in human osteoporosis (9–11). Kuhn et al. and Vahey et al. found qualitative similarities between the mechanical properties of human and canine trabecular bone (11, 12). The long bones of humans and canines also have similar cortical microstructure and vascularization, making the canine an ideal choice for comparative osteological studies regarding the biomechanics of bone (12). Canines have also been established as a strong comparative oncology model for human OS. Canine OS develops spontaneously, and its incidence rate is 10-30x higher than that of human OS (13, 14). Canine OS also shares several biological characteristics with human OS, such as disease presentation, response to treatment, genetic complexity, and histological characteristics (13–15). OS accounts for over 85% of bone tumors in canines, with 95% of patients presenting with spontaneous micrometastases and high levels of pain at time of diagnosis. Cure is achieved in fewer than 15% of dogs (14). Current treatment options for canine OS are similar to those for human OS, the most common involving tumor resection via limb-amputation or limb-salvage surgery and adjuvant chemotherapy. Some dogs may not qualify for limb amputation, and serious complications after limb-salvage surgery such as implant failure and infection occur in 30–70% of patients (16, 17). These similarities between canine and human OS render the dog an ideal model for comparative and translational OS research (13, 14). Given the challenges outlined with current surgical treatment of canine and human OS, novel, non-invasive treatment options for canine OS are sorely needed.

Histotripsy is a non-invasive, non-thermal ablation technique that uses ultrasound to mechanically destroy tissue. Unlike thermally ablative forms of ultrasound, such as high-intensity focused ultrasound (HIFU), histotripsy relies on inertial cavitation; microsecond-long, high-pressure pulses generate microbubbles that rapidly expand and collapse, subjecting cells to high levels of stress and strain and effectively liquefying target tissue (18). This eliminates heat sink effects due to perfusion, which are common in thermal ablation methods and can lead to injury or incomplete treatment (18–20). Since histotripsy relies on mechanical, rather than thermal or biochemical, interactions, it can achieve sub-millimeter precision and discriminate between tissues based on mechanical stiffness (21, 22). Histotripsy has been investigated as a cancer treatment in pre-clinical applications regarding liver (23–25), prostate (26), renal (27), breast (28), brain (29, 30), and pancreatic (31, 32) cancer, as well as in soft tissue sarcomas (33). Extending histotripsy to bone tumors, however, has introduced unique challenges not present in soft tissue, including ultrasound energy reflection from mineralized structures and the highly heterogeneous composition of tumor lesions (34). Despite these challenges, recent studies have demonstrated the safety and feasibility of histotripsy for treating bone tumors through both *in vivo* (21, 35) and *ex vivo* (36) ablation of canine OS tissue. In these studies, histotripsy effectively ablated localized regions of primary OS tumor tissue without causing cell death or damage to normal tissue outside the ablation zone at the same dose. Tissue selectivity has also been demonstrated *in vivo*, in which structures such as the extracellular matrix, blood vessels and nerves within the ablation zone remain structurally intact because their stiffnesses differ from that of the target tissue (36–38). These findings establish a foundation for whole-tumor ablation and the development of effective post-treatment management strategies. However, the genomic instability exhibited by OS leads to highly heterogeneous tumors composed of mixed lytic and sclerotic lesions, making it difficult to effectively target and treat the entire tumor (39). Furthermore, OS frequently invades the cortical bone, compromising its structure and, consequently, its mechanical integrity and strength, which may lead to pathological fracture. The prognosis for OS patients with pathological fractures is poor, involving complications such as fracture hematoma or the spread of micro-metastases (40–42). To address these challenges, it is critical to characterize the effects of histotripsy on the biomechanics of OS tumor-affected bone and how these properties vary spatially throughout the tumor.

Mechanical testing methods such as compression, tensile, nanoindentation, and fracture toughness tests have been used for decades to determine the material properties of bone at the cellular, tissue, and whole bone levels (43, 44). Kuhn et al. and Romanus have characterized the material properties (e.g., elastic modulus, ultimate stress) of canine trabecular and cortical bone via compression and flexural testing, respectively (11, 45). Key properties such as elastic modulus and yield strain derived from the resulting stress–strain curves give insight into a material’s behavior both pre- and post-yield. Coupled with powerful, high-resolution imaging modalities such as μCT, the gold standard for bone microarchitecture assessment, these methods can also facilitate the development of a relationship between μCT intensity, bone mineral density, and elastic modulus, offering insight into structural heterogeneity within a tumor (46–49). The objective of this study was to (1) to determine the effects of histotripsy on the biomechanics of OS tumor-affected and grossly normal bone; and (2) characterize OS tumor heterogeneity. In this study, compression and flexural, namely 3-point bending, tests were utilized to quantify elastic and post-yield mechanical properties of trabecular and cortical bone from *ex vivo* canine limbs, amputated as part of standard of care treatment for OS. The resultant biomechanics and μCT imaging data serve as crucial next steps in developing histotripsy as a safe, effective limb-salvage treatment option for OS.

## Methods

**Figure 1:**
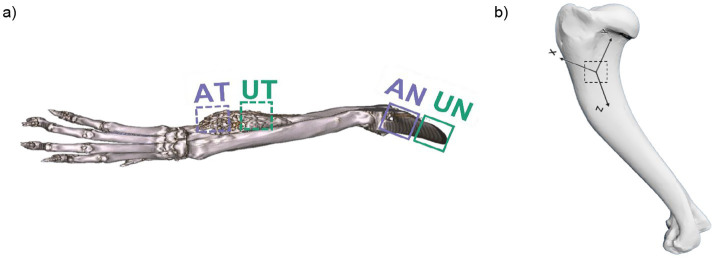
**(a)** Representative 3D model of an OS tumor bearing canine forelimb, generated from CT image data, with anatomical locations identified from which the ablated tumor (AT), unablated tumor (UT), ablated grossly normal (AN) and unablated grossly normal (UN) cortical and trabecular bone specimens were excised – distal radius and distal ulna. **(b)** The primary axis of loading (z-axis) for each bone was identified and utilized to define the remaining principal directions (x- and y-axis) for the trabecular bone specimens—canine humerus.

Amputated limbs (*n* = 10) from canine patients (*n* = 9, 6.6 ± 2.9 years, 33.5 ± 8.36 kg) with primary OS tumors undergoing standard-of-care limb amputation at the Virginia-Maryland Veterinary Teaching Hospital (Blacksburg, VA, USA) were acquired under an approved institutional IACUC protocol (22-091). Patients were enrolled according to predefined criteria, including (1) histologic or radiographic evidence of appendicular OS, (2) no radiographic evidence of pulmonary metastasis, and (3) no previous use of cancer therapy at time of enrollment (35). Patient demographic information is shown in Table 1. Pre-treatment MRI or CT scans were taken of OS-bearing limbs (*n* = 6) to determine the tumor volume for histotripsy ablation, after which patients underwent standard-of-care limb amputation. Imaging modality varied as our clinical team transitioned from CT to MRI for treatment planning and ablation assessment. MRI offers superior soft tissue contrast and provides a more precise assessment of tumor extent and boundaries than CT (50–52). As only the affected limb was surgically removed during standard-of-care treatment and patients remained under clinical care post-operatively, matching healthy (i.e., non-tumor bearing), contralateral limbs were not available for analysis for most patients. In one case, however, both an OS-bearing limb and a grossly normal limb were obtained from the same patient after receiving owner consent for limb excision after euthanasia. Tumor tissue specimens were collected immediately following limb amputation for histological confirmation of OS. Histotripsy treatments were performed using a custom 32-element 500 kHz histotripsy transducer within 48 h post-amputation to minimize tissue degradation and to preserve biomechanical and histological integrity. Pulses of 1–2 cycles (pulse duration: 2–4 μs) were applied to the center of OS tumor samples and a matching volume of grossly normal bone at a pulse repetition frequency of 500 Hz with 1,000 pulses/point, as described in previous studies regarding histotripsy ablation of canine OS (21, 36). Grossly normal bone was identified adjacent to the tumor, often from the opposing end (proximal vs. distal) or in neighboring bones across a joint (tibia vs. femur). Histotripsy treatments were guided in real-time using an ultrasound imaging probe (Model C5-2, Analogic Corporation, Peabody, MA, USA), and the cavitation cloud threshold for each sample was determined by visualizing the cavitation cloud and gradually increasing the pressure at the focus until a consistent cloud was observed on real-time ultrasound imaging (36, 37). Targeted volumes ranged from 10.1 to 24.0 cm^3^ and peak negative pressures, applied ~20% above the cavitation threshold, ranged from 26.5 to 35.7 MPa. Post-treatment of the tumor-bearing limb, the affected bone was resected and split along the anatomical axis for biomechanical and histological analysis. Specimens for biomechanical analysis were stored at −20 °C until testing. Given that the tissue was not fixed, time between amputation and storage was limited to 48 h post-amputation. Bone tissue from ablated tumor (AT, *n* = 9), ablated normal bone (AN, *n* = 10), unablated tumor (UT, *n* = 7), and unablated normal bone (UN, *n* = 16) were then extracted for biomechanical testing (Figure 1a). Treated and untreated tumor specimens were collected from different limbs, as the goal of the histotripsy treatment was to ablate as much of the tumor as possible.

**Table 1:** Demographic information for all patients, including anatomical locations from which cortical and trabecular tissue specimens were extracted.

Demographic information						Tissue taken from (*n* = trabecular, cortical)			
Patient	Breed	Age (years)	Gender	Weight (kg)	Tumor location	UN	AN	UT	AT
1	Greyhound	8	FS	30.7	Distal radius	Proximal humerus (*n* = 0, 3)	Proximal radius (*n* = 0, 3)		Distal radius (*n* = 0, 4)
2	Boxer	3	MN	30.0	Medial humeral condyles	Proximal humerus (*n* = 0, 4)	Distal humerus (*n* = 0, 3)		Medial humeral condyles (*n* = 0, 3)
3	Golden Retriever	1	MN	25.0	Distal ulna	Distal humerus (*n* = 0, 4)	Proximal humerus (*n* = 0, 3)		Distal ulna (*n* = 0, 3)
4	Cane Corso	9	FS	50.4	Distal humerus			Distal humerus (*n* = 7, 10)	
5	Mixed breed	9	FS	25.9	Proximal tibia			Proximal tibia (*n* = 0, 8)	
6	Boxer	5	FS	26.1	Distal radius		Proximal ulna (*n* = 8, 7)		
7^†^	Golden Retriever	9	FS	40.0	Proximal humerus	Proximal tibia (*n* = 10, 0)	Distal radius (*n* = 2, 0)		Proximal humerus (*n* = 9, 12)
8	Golden Retriever	7	MN	35.5	Distal humerus		Distal humeral condyles (*n* = 5, 0)		
9	Great Dane	8	MN	38.3	Distal radius	Distal humerus (*n* = 6, 0)			

† Both an OS tumor-bearing forelimb and a grossly normal hindlimb were excised from this patient.

Cubic specimens (7.5 mm × 7.5 mm × 7.5 mm) were cut from the trabecular bone sections using an IsoMet low-speed precision saw under constant irrigation. The edges of the cubes were cut parallel to the primary axis of loading (Z) shown in Figure 1b. These samples were placed centrally between two steel compression disks mounted to an MTS Insight 10 materials testing system. Preconditioning cycles (*n* = 5) ranging from 20 to 100 N were applied at a loading rate of 0.01 mm/s to ensure full engagement of the sample surface with the compression platens before testing. Then, repeat uniaxial compressive loading (*n* = 3) was performed at a strain rate of 1%/s with a 20 N preload in three anatomical directions (superior–inferior, medial-lateral, and anterior–posterior) until just under 3% strain to assess tissue anisotropy and to determine the compressive modulus of each sample. Compression to failure (10% strain) along the primary axis of loading (Z-axis, Figure 1b) was performed to determine the yield and ultimate compressive stresses of each sample. Rectangular beam specimens (15 mm × 2 mm × 1 mm) of cortical bone were cut from the diaphysis of each tissue region along the longitudinal bone axis. Specimens were subjected to 3-point bending until failure (ElectroForce 3200) with a constant displacement rate of 0.05 mm/min and a support span of 12 mm to evaluate pre- and post-yield cortical bone properties.

Trabecular, humeral UT specimens were micro-CT scanned (Bruker SkyScan 1172) prior to mechanical testing at a voxel resolution of 17.5 × 17.5 × 17.5 μm (60 kV, 167 μA) for assessment. Specimens were placed in conical test tubes, submerged completely in phosphate buffer solution (PBS, pH 7.4, Sigma Aldrich), and mounted in a cylindrical specimen holder stabilized with a foam mold. Regions of interest (ROIs) were chosen to be uniform in size and space across specimens. Three-dimensional reconstructions of each specimen were obtained from the scans using scanner software and visualized in 3D Slicer. Binarized segmentation masks were created using 3D Slicer’s Otsu thresholding method to identify mineralized bone from background. These masks were then used to calculate the trabecular bone volume fraction (BV/TV) of each specimen.

All trabecular and cortical specimens were stored at −20 °C prior to testing and were kept hydrated with sprays of deionized water during testing to maintain mechanical integrity. All testing was performed within 2 months of limb amputation and *ex vivo* histotripsy treatment. For compression tests, stress (*σ*) and strain (*ε*) were calculated according to Equations 1, 2 (53):


$$\sigma =\frac{F}{A}$$ (1)

$$\varepsilon =\frac{\mathrm{\Delta L}}{L_{0}}$$ (2)

Where *F* is the applied load, *A* is the cross-sectional area of the specimen, *ΔL* is the crosshead displacement, and *L_0_* is the gauge length of the specimen. For 3-pt bending tests, stress and strain were calculated according to Equations 3, 4 (53):


$$\sigma =\frac{3\mathrm{FL}}{2bd^{2}}$$ (3)

$$\varepsilon =\frac{6\mathrm{Dd}}{L^{2}}$$ (4)

Where *L* is the span length, *D* is the crosshead displacement at the midpoint of the specimen, and *b* and *d* are its width and thickness, respectively. Compressive and bending moduli were derived from the linear region of the stress–strain curves, consistent with Hooke’s law, which relates stress to strain in elastic deformation (53). Ultimate strength was also determined for both trabecular and cortical specimens to assess the impact of histotripsy on tissue strength, while compressive and bending moduli were used to evaluate tissue stiffness.

All statistical analyses were performed in Python 3.9 (statsmodels 0.13.2, scipy 1.7.3). A linear mixed effects model was used to assess the impact of loading direction and tissue on the pre-yield properties of the trabecular bone specimens. Due to the nonparametric nature of the collected data, a Kruskal-Wallis test was used to assess elastic and post-yield behavior of both the trabecular and cortical bone specimens; where necessary, Dunn’s test was performed as a post-hoc measure to compare data between groups. The alpha value was set at 0.05 and *p*-values were adjusted for multiple comparisons. Coefficient of variation (CV), calculated as the ratio of the standard deviation to the mean, was also determined for each sample to assess the reproducibility of repeated uniaxial loading tests.

## Results

**Figure 2:**
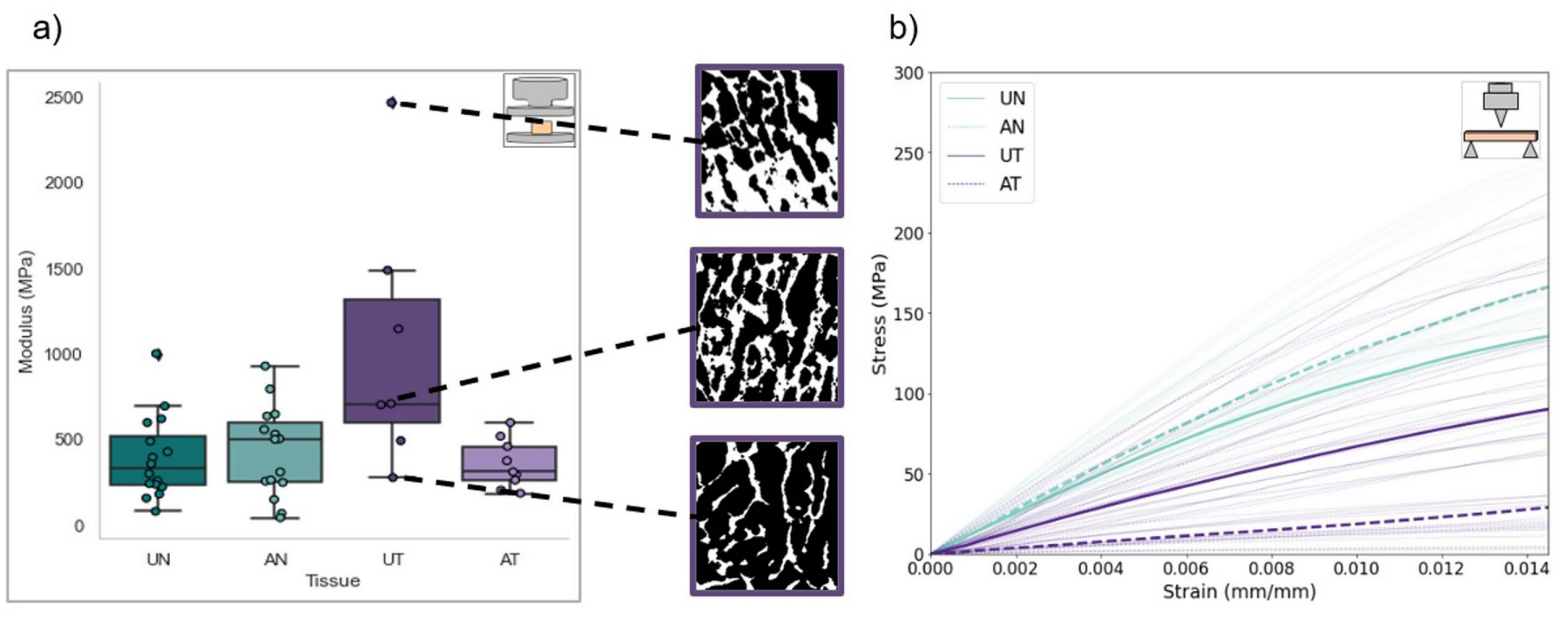
**(a)** Elastic modulus distribution for **(a)** cortical bone and **(b)** trabecular bone specimens. μCT slices of select UT specimens prepared for compression testing, demonstrating the range in bone volume fraction of the trabecular network.

**Figure 3:**
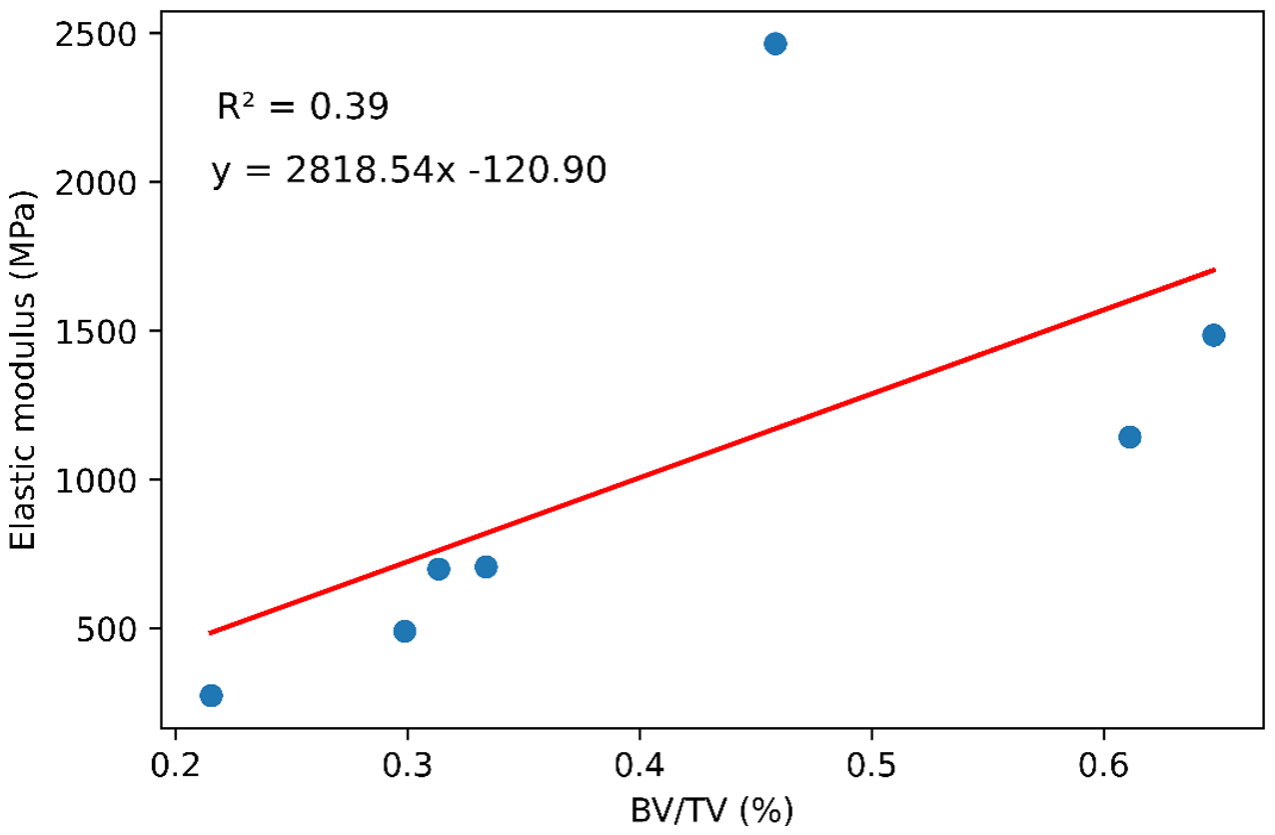
Relationship between BV/TV and elastic modulus of trabecular UT samples.

The mechanical properties of 47 trabecular cubic specimens were analyzed. Cube dimensions varied by <2% in all directions. Mean CVs from repeat pre-yield compression tests for each group ranged from 4.6 to 13.5%, with the majority of specimens (85%) exhibiting CVs below 15%. Elastic modulus was significantly higher in UT specimens than in UN, AN, and AT specimens (*p* = 0.0011, *p* = 0.0026, *p* = 0.0022, respectively). No significant differences were found in post-yield properties between groups; however, UT specimens exhibited a higher value and range regarding ultimate strength, and a higher range regarding yield stress and strain than the other groups (Figure 2a; Table 2). The BV/TV of these specimens was measured, with a mean of 0.411 ± 0.166. μCT analysis of UT specimens revealed a wide range of trabecular densities, highlighting high levels of tumor heterogeneity and a linear, albeit weak (*R*^2^ = 0.39), trend between UT BV/TV and elastic modulus (Figure 3). Regarding anisotropy, stiffness was often higher in the primary loading direction for UN and UT specimens, but no significant differences were found within the three loading directions between unablated tissue groups. Stiffness in the primary loading direction for AN and AT specimens was significantly higher than in the other two directions (*p* = 0.035, *p* < 0.001, respectively).

**Table 2:** Material property data for trabecular and cortical bone specimens.

Mechanical test	Property	UN (*n* = 16)	AN (*n* = 10)	UT (*n* = 7)	AT (*n* = 9)	(*p*)
Compression	Ultimate strength (MPa)	15.1 ± 8.06	15.0 ± 11.7	33.0 ± 18.3	14.9 ± 7.00	0.089
	Compressive modulus (MPa)	389 ± 241	427 ± 264	728 ± 446	354 ± 143	≪0.001*
	Yield strength (MPa)	10.0 (9.40)	13.5 (12.5)	13.3 (21.4)	9.58 (9.30)	0.461
	Yield strain (%)	2.66 (0.631)	2.42 (1.21)	2.79 (2.10)	3.01 (0.691)	0.290

	Property	UN (*n* = 11)	AN (*n* = 16)	UT (*n* = 18)	AT (*n* = 22)	(*p*)
Bending	Ultimate strength (MPa)	189 (99.1)	214 (63.6)	116 (109)	115 (114)	≪0.001*
	Bending modulus (GPa)	12.2 (6.85)	12.0 (4.91)	6.57 (6.47)	4.95 (7.07)	≪0.001*
	Elastic work (J)	1.05 (0.401)	1.11 (0.341)	1.05 (0.766)	0.992 (1.07)	0.956
	Toughness (MJ/m^3^)	3.93 (1.97)	8.35 (5.74)	2.80 (2.27)	2.23 (3.22)	≪0.001*

Data reported as mean ± standard deviation or median (IQR). * indicates statistical significance (*p* < 0.05).

The mechanical properties of 67 cortical beams were analyzed. Beam dimensions varied by <3% in all directions. Ultimate strength, taken from the point of highest load, was significantly higher in grossly normal bone (UN and AN) groups than in tumor tissue (UT and AT) groups. No significant differences were found within normal bone groups or within tumor tissue groups. Elastic modulus followed a similar trend; normal bone specimens were significantly stiffer than tumor tissue specimens, and no differences were found within normal bone groups or within tumor tissue groups (Figure 2b; Table 2). Toughness values were significantly higher in AN specimens than in AT and UT specimens (*p* = 0.003, 0.003, respectively).

## Discussion

The aim of the proposed study was to evaluate the biomechanical effects of histotripsy on OS tumor-affected and normal bone. This study successfully demonstrated that histotripsy ablation at clinical doses does not detrimentally affect the mechanical properties of normal bone, as no significant differences were observed between histotripsy-ablated and unablated normal bone. Similarly, the lack of differences in the mechanical properties of ablated and unablated tumor-affected cortical bone suggests that histotripsy treatment does not further compromise the structural integrity of tumor-affected bone. For standard-of-care surgical approaches, resection typically includes 2–3 cm of surrounding bone and soft tissue to ensure complete excision of malignant cells (54, 55). In this study, histotripsy was applied without a margin; however, future applications may incorporate a conservative margin to effectively target microscopic disease, similar in concept to those implemented in radiation therapy (56). These results indicate that such a treatment margin could be feasible without further compromising bone integrity. Together, these findings support the histotripsy’s potential as an effective treatment for OS tumors that does not increase risk of fracture in the affected limb. These tissue-selective features offer the potential for effective tumor ablation near critical structures, which has been a problem for other therapeutic interventions such as thermal ablation and radiation; thermal ablation methods such as HIFU often expose tissue to temperatures exceeding 50 °C, leading to cell lysis, protein denaturation, and necrosis (57, 58), while therapeutic doses of radiation have been reported to modify the histology of bone, damage its structural integrity, and delay healing (59–61). The vessel-sparing and duct-sparing features of histotripsy are well established in the treatment of liver cancer, enabling safer and more effective tumor ablation compared to thermal-based approaches in both preclinical and clinical studies (23–25). The findings in this study suggest that histotripsy’s tissue-selective and bone-sparing properties can similarly be used for the treatment of OS. By effectively ablating tumors without increasing fracture risk, histotripsy represents a promising alternative for addressing the limitations of existing therapeutic modalities.

Histotripsy’s ability to preserve the mechanical integrity of bone is highly significant given the already-compromised nature of OS-affected bone. The altered mechanical integrity of tumor-affected bone may increase the risk of pathological fracture, which occurs in 5–10% of patients and is a significant clinical concern (62, 63). Patients who present with pathological fractures have an increased risk of local recurrence, spread of micrometastases, and decreased survival rates (64, 65). This is further exacerbated by the highly variable mechanical phenotype of OS-affected bone, caused by its heterogenous nature. For example, lytic regions may have a catabolic effect, promoting bone degradation and structural weakening, while sclerotic regions induce an anabolic response, producing abnormally dense bone that is stiffer but potentially more brittle (66). As a result, the mechanical response to histotripsy may differ depending on the tumor phenotype; highly lytic lesions would likely require alternative testing approaches as the tissue may be too fragile for compression testing, while mixed or sclerotic lesions may respond differently to acoustic energy deposition. This variability complicates risk assessment and highlights the importance of optimizing histotripsy strategies to ensure effective ablation across varied tumor compositions. Future work will incorporate high-resolution μCT-based lesion classification to quantify compositional differences and correlate them with mechanical performance and treatment outcomes, ultimately enabling patient-specific treatment strategies across tumor subtypes.

In the present study, mechanical testing results demonstrated that cortical bone properties in normal bone specimens were slightly lower than previously reported values (67–70). This may be due to unintended trabecular involvement in samples taken from the endocortical surface, given the limited tissue availability in the distal segments of the sampled long bones. For the tumor tissue, no comparable mechanical properties for OS tissue are available in the literature, but the reduced mechanical properties observed in the OS tumor tissue relative to the grossly normal bone in these data reinforces the observed phenomenon of compromised structural properties of OS tumor tissue even prior to histotripsy treatment. Post-yield trabecular specimen properties are in line with previously published literature values for all groups (UN, AN, AT) except UT; Kang et al. found the elastic modulus and ultimate load of a healthy canine humeral head, which several of the specimens in this study were excised from, to be 350 ± 171 MPa and 225 ± 74 N, respectively (71). The large range in trabecular properties for UT specimens reported in this study implies a significant degree of structural and material heterogeneity, which is characteristic of OS tumor tissue (72–74). Evaluation of the structural properties, namely BV/TV, of select trabecular UT specimens also revealed a wide range of values, some of which fall above those previously published in literature for grossly normal canine trabecular bone; BV/TV of canine trabecular bone has been shown to range from approximately 15–45% when sampled from lumbar spine and femoral anatomical locations (75–77). The higher BV/TV values reported here may demonstrate OS-induced osteoblastic activity, leading to disruption of trabecular architecture and the formation of high-density areas of sclerosis, or unintended cortical involvement. Regarding anisotropy, significant differences in elastic modulus between loading directions were only observed within ablated tissue groups. These findings may imply that histotripsy contributes to a stiffening effect in the primary loading direction; however, confounding factors such as donor demographics and small sample size cannot be excluded. Future studies should assess the mechanical properties of histotripsy-treated bone in multiple loading directions to determine whether histotripsy affects bone anisotropy and directional load dependence.

This study has important limitations that should be considered when interpreting findings. The clinical nature of this pilot study prevented complete control over key variables such as tissue donor characteristics (age, weight, sex, etc.), tumor characteristics, anatomical sampling location, and sample size. Small and uneven group sizes may have limited statistical power and the generalizability of results, highlighting the need for larger, more balanced cohorts. Variations in donor characteristics and tumor location may have also contributed to differences in baseline mechanical properties; future work should consider stratification by age, body weight, or tumor location to minimize these effects. Additionally, because patients remained under clinical care following standard-of-care amputation, matched contralateral limbs were not available for use as controls. As such, observed differences between groups may partly reflect these confounding factors. Trabecular bone architecture is also highly heterogeneous, making this tissue difficult to mechanically analyze without accompanying robust, density-calibrated image analysis (74, 78, 79). To address these limitations, controlled, dose-dependent studies are needed to evaluate the biomechanical effects of histotripsy on healthy and OS-affected tissue, establish treatment guidelines, and determine safe thresholds for ablation that preserve surrounding, healthy structures. Further characterizing the relationship between tumor heterogeneity and histotripsy dose is also imperative for ensuring complete tumor ablation and maximizing therapeutic safety and efficacy.

Histotripsy is currently being explored in combination with chemotherapy for liver tumors, such as colorectal liver metastases (80) and preclinical studies are investigating its integration with immunotherapy and chemotherapy across various cancer types (81, 82). Although these studies are not yet osteosarcoma-specific, they establish a precedent for histotripsy’s potential as a multimodal therapy. As a neoadjuvant therapy, histotripsy could reduce tumor burden prior to surgery and improve surgical outcomes. As an adjuvant therapy, histotripsy could be applied to post-surgical tumor to ablate residual disease, potentially reducing local recurrence. In combination with immunotherapy, histotripsy may enhance immune activation through the release of tumor antigens, supporting its own anti-tumor abscopal response (83).

## Conclusion

In this study, the effects of histotripsy on the biomechanical properties of tumor tissue and grossly normal bone were evaluated, demonstrating its potential as a safe, bone-sparing treatment option for OS. Both trabecular and cortical bone specimens were mechanically tested to failure via compression and 3-pt bending, respectively. No significant differences were found in mechanical properties between ablated and unablated normal bone groups, supporting the hypothesis that clinical doses of histotripsy do not detrimentally affect the biomechanical properties of normal cortical or trabecular bone. Additionally, no significant differences were found in mechanical properties between ablated and unablated tumor groups, indicating that histotripsy treatment does not further compromise the biomechanical integrity of OS tumor-affected bone. Trabecular, unablated tumor specimens showed a wide range in trabecular density and mechanical properties, highlighting the heterogeneity of OS tumors and the difficulties that may arise while attempting to treat a whole tumor volume. Ultimately, the results of the current work will aid in the development and advancement of histotripsy as an effective, non-invasive, bone-sparing, and patient-specific treatment option for clinical OS.

## Data Availability

The raw data supporting the conclusions of this article will be made available by the authors, without undue reservation.
